# TRODO: A public vehicle odometers dataset for computer vision

**DOI:** 10.1016/j.dib.2021.107321

**Published:** 2021-08-31

**Authors:** Kaouther Mouheb, Ali Yürekli, Burcu Yılmazel

**Affiliations:** Department of Computer Engineering, Eskişehir Technical University, Eskişehir 26555, Turkey

**Keywords:** Computer vision, Image processing, Digit recognition, Odometer, Mileage reading

## Abstract

In the field of transportation and logistics, smart vision systems have been employed successfully to automate various tasks such as number-plate recognition and vehicle identity recognition. The development of such automated systems is possible with the availability of large image datasets having proper annotations. The TRODO dataset is a rich-annotated collection of odometer displays that can enable automatic mileage reading from raw images. Initially, the dataset consisted of 2613 frames captured in different conditions in terms of resolution, quality, illumination and vehicle type. After data pre-processing and cleaning, the number of images was reduced to 2389. The images were annotated using the CVAT image annotation tool. The dataset provides the following information for each frame: the type of odometer (analog or digital), the mileage value displayed on the odometer, the bounding boxes of the odometer, and the digits and characters displayed on the screen. Combined with machine learning and artificial intelligence, the TRODO dataset can be used to train odometer classifiers, digit recognition and number reading models from odometers and similar types of displays.

## Specifications Table

**Table:** 

Subject	Computer Vision and Pattern Recognition
Specific subject area	Object detection, Region-of-interest detection, and Digit recognition
Type of data	Images (JPG files)
	Annotations in the Pascal VOC 1.1 format [1] (XML files)
	Ground truth (JSON file)
How data were acquired	The images were acquired by capturing vehicle boards using smartphone cameras of different qualities. During the annotation phase, the Computer Vision Annotation Tool^1^ (CVAT) has been used.
Data format	Raw (images)
	Processed (annotations and ground truth)
Parameters for data collection	The images were captured with varying parameters of illumination, resolution, and rotation. Furthermore, the odometer displays belong to different vehicles in different models.
Description of data collection	The dataset can be described as a collection of 2389 annotated images of analog and digital odometer displays captured using smartphone cameras.
Data source location	Institution: Eskişehir Technical University
	City: Eskişehir
	Country: Turkey
	Latitude and longitude for collected data: 39.766193, 30.526714
Data accessibility	Repository name: Mendeley Data
	Data identification number: https://doi.org/10.17632/6y8m379mkt.1
	Direct URL to data: https://doi.org/10.17632/6y8m379mkt.1

## Value of the Data

• The TRODO dataset presents annotated images of odometer displays that belong to different types of vehicles. The images are provided by Marketyo,^2^ an omni-channel marketing and e-commerce company serving in Turkey. Accordingly, the dataset can be considered as real-world data of an active product delivery system.
• The TRODO dataset offers a great research opportunity for miscellaneous computer vision tasks, such as object detection and digit recognition. Researchers working in the field can use the dataset to train, validate, and test novel or existing supervised machine learning approaches.
• In the fields of vehicle insurance and logistics, the TRODO dataset can be utilized to build smart vision systems for mileage reading from odometer images. It is also possible to extend the application areas of the dataset by transfer learning.
• The TRODO dataset is valuable for the development of automated techniques to extract latent information available in mechanical or digital odometer-like screens.

## 1 Data Description

The TRODO dataset is a collection of annotated odometer images that belong to several vehicles operating in the product delivery network of Marketyo. The dataset contains a total of 2389 raw images in two types, analog and digital. Each of these images is provided with proper annotations of an odometer region and recognizable characters in this region. Furthermore, the ground truth of actual odometer types and mileages are also provided for the purpose of model evaluation in classification tasks.

As a publicly available distribution in a Mendeley Data repository,^3^ the TRODO dataset is organized in three main folders:

i. *images* folder contains the raw images.
ii. *pascal voc 1.1* folder contains the annotations in the Pascal VOC 1.1 format.
iii. *ground truth* folder contains the actual values of odometer types and mileages.

For the rest of this section, we follow the structure presented above to describe further details about the dataset.

### 1.1 Images

**Fig. 1:**
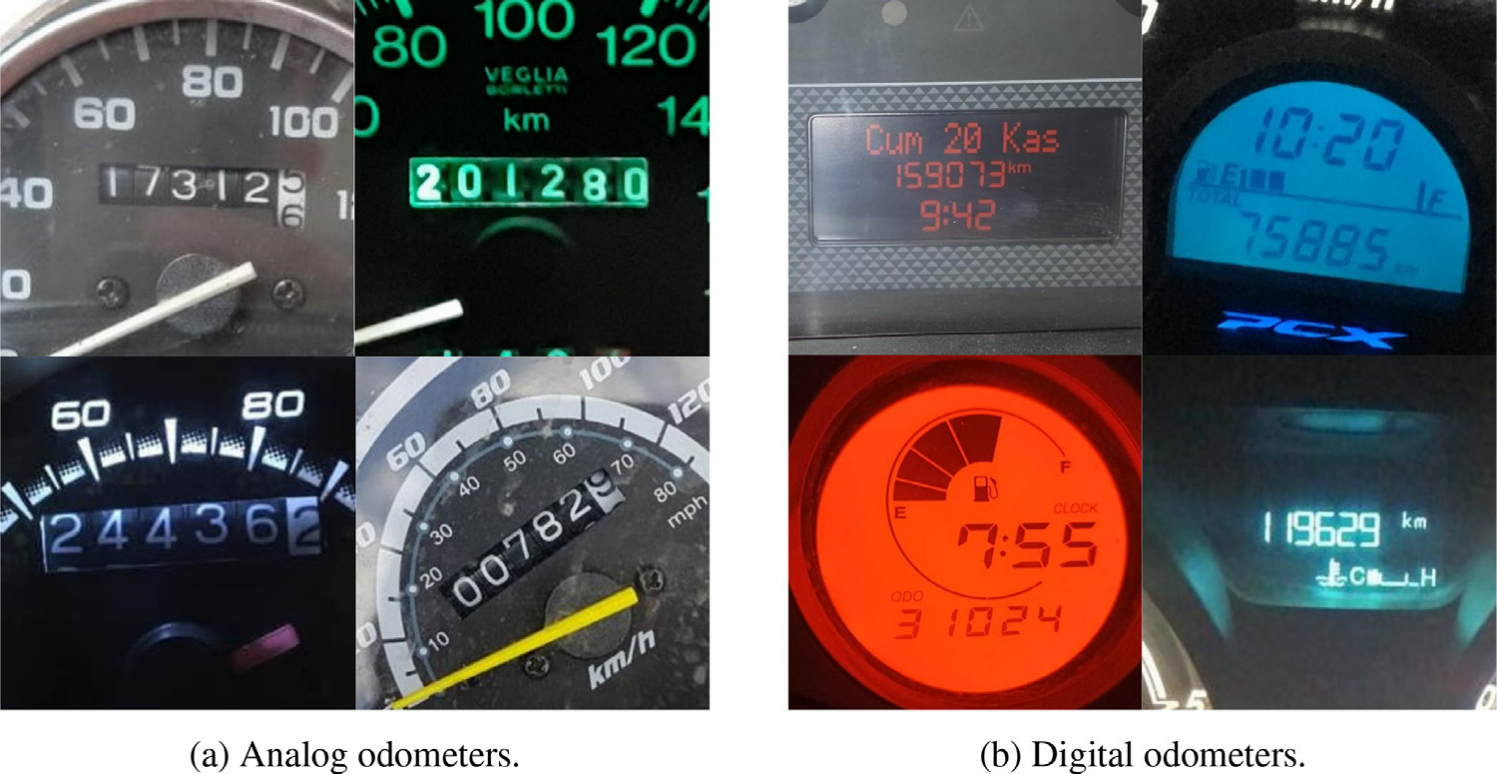
Samples of raw odometer images in analog and digital types.

The *images* folder contains raw odometer images in JPG format. There exist a total number of 2389 images in this folder, 858 of which are analog odometers and 1531 are digital odometers. In Fig. 1, we present some samples of both odometer types. As illustrated in the figure, the odometers may include various information such as the date, time, and air temperature as well as the total mileage of the vehicles.

**Fig. 2:**
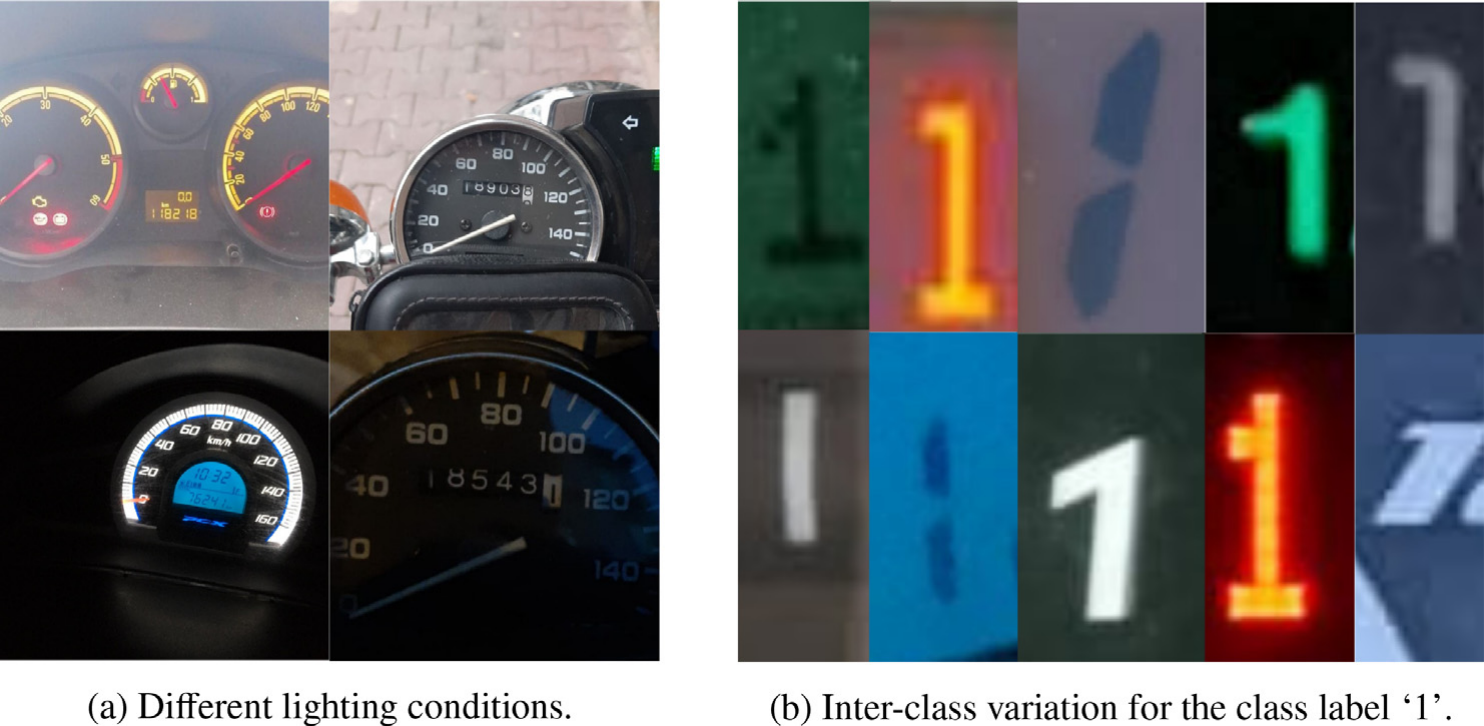
Samples illustrating different lighting conditions and inter-class variation in odometer images.

The images were captured using smartphone cameras with variable resolutions with an average of 886×1173 pixels. Due to the varying conditions of image capturing, the TRODO dataset involves a high diversity of lighting conditions ranging from very high (daylight) to low (night illumination), as presented in Fig. 2a. Moreover, the odometer images belong to different vehicles, which introduces a large inter-class variation to the dataset. Fig. 2b exemplifies this variation; all the partial image regions represent the digit ‘1’ in different styles.

### 1.2 Annotations

The *pascal voc 1.1* folder contains the corresponding annotations of the images in the Pascal VOC format. Each image in the dataset can be associated with an XML file in this folder using the file name of the image. The XML files (2389 files in total) include the objects detected in the frames with their bounding box coordinates. Some auxiliary files, such as the list of image names (‘default.txt’) and bounding box color codes for class labels (‘labelmap.txt’), can be also found in this folder.

**Fig. 3:**
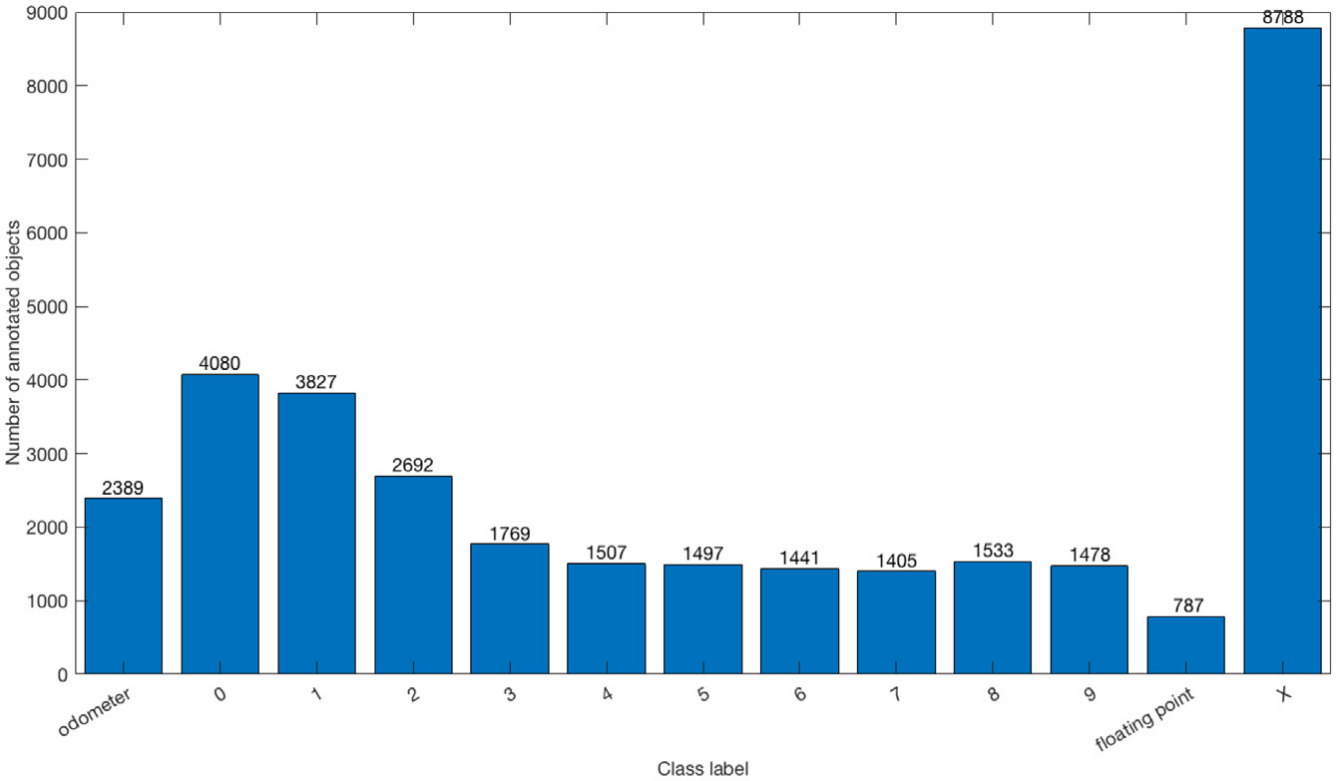
The distribution of annotated objects in the TRODO dataset in terms of class labels.

The TRODO dataset contains 13 categories of annotations: the region of odometer display, the instances of 10 digits visible on the annotated region, the floating point, and the special category ‘X’ that represents non-digit characters. In Fig. 3, we present the distribution of the annotated objects within the dataset in terms of these class labels. As shown in the figure, the digits ‘0’, ‘1’, and ‘2’ appear more frequently in the odometer displays than the other digits. The frequencies of the other digits are highly close to each other.

### 1.3 Ground truth

In supervised machine learning, the availability of class labels is of special importance [2]. Thus, along with the images and their corresponding annotations, we also provide the ground truth of odometer types and mileages. The *ground truth* folder contains a single JSON file (‘groundtruth.json’) that stores this information. Each entry in this file consists of the image file name, the odometer type, and the total mileage. Researchers working in computer vision can use this file to train, validate, test, and evaluate models for various classification tasks.

## 2 Experimental Design, Materials and Methods

In cooperation with Marketyo, we acquired raw JPG images of odometer displays from the company’s product delivery network. The original images were captured from different vehicles (cars and motorcycles) using smartphone cameras. Initially, the collection had 2613 candidate images. On this collection, we performed data pre-processing and data annotation steps as described in the rest of this section.

**Fig. 4:**
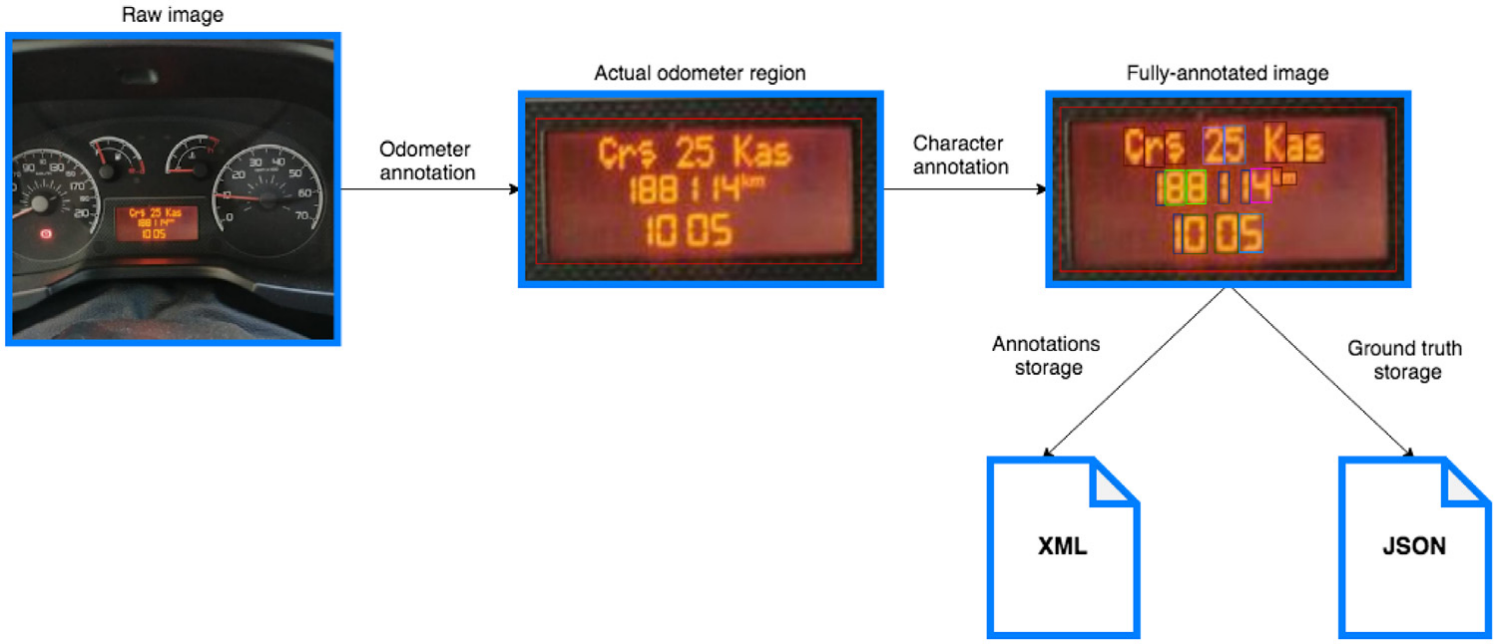
The workflow of data annotation for the TRODO dataset.

### 2.1 Data pre-processing

Once we acquired a collection of odometer images, we proceeded to the data pre-processing step that involves two main tasks: *(i)* validating the images and *(ii)* building a ground truth from the valid images.

Firstly, the images that do not meet certain criteria, such as quality, resolution, and clarity, or that contain sensitive information (e.g., face reflection of the employee capturing the image or any contact information), were eliminated. Then, by applying the inter-annotator agreement [3], the odometer types and total mileages on displays were determined as the ground truth. Consequently, the data pre-processing step resulted in a final set of 2389 odometer images with a valid ground truth.

### 2.2 Data annotation

In order to make the TRODO dataset appropriate to train and evaluate machine learning models, we carried out an annotation process on all 2389 odometer images. During this process, we used the CVAT, which a popular online platform for image annotation, to obtain a set of fully-annotated images.

The process to label the images in the dataset can be expressed as follows:

• The odometer displays were segmented by drawing a bounding box enclosing the display (LCD screens from digital odometers and mechanical meters from analog odometers).
• The boxes enclosing each individual character inside the actual odometer region were drawn. Then, these boxes were labeled with corresponding class labels. Notably, along with the 10 digits and the floating point, we also use a special class ‘X’ to identify other kinds of information (e.g., date, radio channel, and temperature) than mileage.

Fig. 4 illustrates the workflow of the data annotation step described above. Afterward, we assembled raw images, annotations, and ground truth as a single, complete package that will be published in a Mendeley Data repository.

## Ethics Statement

The author duly adhere to ELSEVIER ‘Ethics in Research & Publication’ policy.

## CRediT authorship contribution statement

**Kaouther Mouheb:** Methodology, Formal analysis, Data curation, Writing – original draft. **Ali Yürekli:** Resources, Methodology, Data curation, Writing – review & editing. **Burcu Yılmazel:** Resources, Data curation, Writing – review & editing, Supervision, Project administration.

## Declaration of Competing Interest

The authors declare that they have no known competing financial interests or personal relationships which have, or could be perceived to have, influenced the work reported in this article.

## Footnotes

3 https://doi.org/10.17632/6y8m379mkt.1

## Footnotes

1 https://cvat.org

2 https://www.marketyo.com
